# Optimization by Box–Behnken design for environmental contaminants removal using magnetic nanocomposite

**DOI:** 10.1038/s41598-024-57616-8

**Published:** 2024-03-23

**Authors:** Luis Buenaño, Eyhab Ali, Ahmed Jafer, Shaima Haithem Zaki, Fathi Jihad Hammady, Salima Baqir Khayoun Alsaadi, Manal Morad Karim, Montather F. Ramadan, Alaa A. Omran, Ahmed Alawadi, Ali Alsalamy, Ali Kazemi

**Affiliations:** 1 Facultad de Mecánica, Escuela Superior Politécnica de Chimborazo (ESPOCH), Riobamba, 060155 Ecuador; 2 Al-Zahraa University for Women, Karbala, Iraq; 3 Department of Radiology and Sonar, Al-Manara College for Medical Sciences, Amarah, Maysan Iraq; 4 Department of Anesthesia Techniques, Al-Noor University College, Nineveh, Iraq; 5 Department of Medical Engineering, Mazaya University College, Nasiriyah, Dhi Qar Iraq; 6 Department of Medical Engineering, Al-Hadi University College, Baghdad, 10011 Iraq; 7 College of Pharmacy, National University of Science and Technology, Nasiriyah, Dhi Qar Iraq; 8 College of Dentistry, Al-Ayen University, Nasiriyah, Dhi Qar Iraq; 9 Department of Medical Engineering, AL-Nisour University College, Baghdad, Iraq; 10 College of Technical Engineering, The Islamic University of Najaf, Najaf, Iraq; 11 College of Technical Engineering, The Islamic University of Al Diwaniyah, Al Diwaniyah, Iraq; 12 College of Technical Engineering, The Islamic University of Babylon, Babylon, Iraq; 13 College of Technical Engineering, Imam Ja’afar Al‐Sadiq University, Baghdad, Al‐Muthanna 66002 Iraq; 14 School of Public Health, Tehran University of Medical Sciences, Tehran, Iran

**Keywords:** Box–Behnken design, Dye, Heavy metals, Removal, Response surface methodology, Environmental sciences, Chemistry

## Abstract

In this study, a CoO–Fe_2_O_3_/SiO_2_/TiO_2_ (CIST) nanocomposite was synthesized and utilized as an adsorbent to remove methylene blue (MB), malachite green (MG), and copper (Cu) from aqueous environments. The synthesized nanocomposite was characterized using field emission scanning electron microscopy (FE-SEM), Fourier-transform infrared spectroscopy (FTIR), thermogravimetric analysis (TGA), and X-ray diffraction (XRD). Input parameters included pH (3–10), contact time (10–30 min), adsorbent amount (0.01–0.03 g), and pollutant concentration (20–60 mg L^−1^). The effects of these parameters on the removal process efficiency were modeled and optimized using the response surface methodology (RSM) based on the Box–Behnken design (BBD). The RSM-BBD method demonstrated the capability to develop a second-degree polynomial model with high validity (R^2 ^˃ 0.99) for the removal process. The optimization results using the RSM-BBD method revealed a removal efficiency of 98.01%, 93.06%, and 88.26% for MB, MG, and Cu, respectively, under optimal conditions. These conditions were a pH of 6, contact time of 10 min, adsorbent amount of 0.025 g, and concentration of 20 mg L^−1^. The synthesized adsorbent was recovered through five consecutive adsorption–desorption cycles using hydrochloric acid. The results showed an approximately 12% reduction from the first to the seventh cycle. Also, MB, MG, and Cu removal from real water samples in optimal conditions was achieved in the range of 81.69–98.18%. This study demonstrates the potential use of CIST nanocomposite as an accessible and reusable option for removing MB, MG, and Cu pollutants from aquatic environments.

## Introduction

In recent decades, water quality and (industrial and urban) wastewater have become a critical and vital concern worldwide. Regarding the rapid growth of industry and technology, industrial production and human consumption have increased, leading to a rise in the concentration of pollutants in water sources^1,2^.

Meanwhile, heavy metals such as Cu are recognized as well-known pollutants in water^3^. Due to its toxicity and long-term environmental stability, Cu is highly detrimental and can threaten the health of humans and aquatic animals^4,5^. Therefore, from a public health and environmental conservation perspective, it is essential to eliminate and reduce the concentration of this metal in water.

Furthermore, dyes such as MG and MB have been identified as water pollutants in both groundwater and surface water^6,7^. In this respect, MG and MB dyes are extensively used in various industries, including textile, dyeing, and printing. A major challenge with these compounds is that when released into aquatic environments, they act as both surface and groundwater pollutants, thereby causing harm to ecosystems and public health^8,9^. Hence, removing and purifying these compounds from water are essential for preserving water resources.

Various methods (e.g., aerobic and anaerobic digestion, coagulation, advanced oxidation, surface adsorption, and membrane filtration) have been employed to treat wastewater^10–14^. Each method has different removal efficiencies, advantages, disadvantages, and investment and operational costs. Considering its initial costs, simplicity in design, ease of operation, and insensitivity to toxic substances, surface adsorption proves to be an effective wastewater treatment process^15,16^. Additionally, surface adsorption can generate high-quality effluents free from undesirable substances (e.g., ozone and free radicals)^17,18^.

While surface adsorption is naturally potent, its mass transfer resistance is widely limited due to the size of adsorbent particles. This limitation can be overcome by leveraging nanotechnology^19^. Nanotechnology, aimed at mitigating the adverse effects of environmental pollutants, can be considered an environmentally friendly management solution^20^. Nanomaterials possess key physical and chemical characteristics such as high surface-to-volume ratio, high reactivity, better catalytic potential, and resistance to internal diffusion. These attributes can contribute to improving existing wastewater treatment technologies^21,22^.

In the last decade, magnetic nanomaterials (MNPs) have been used to treat water containing heavy metals, pesticides, and dyes. Magnetic nanoparticles, including magnetite (Fe_2_O_3_), maghemite (Fe_3_O_4_), and zero-valent iron at the nanoscale, have many applications in medicine, water treatment, and biology^23,24^. For instance, Shokri and Sanavi Fard^25^ used Fe_2_O_3_/SiO_2_ to remove chlorophenol. Shokri and Sanavi Fard concluded that the maximum amount of removal occurred at a mass ratio of H_2_O_2_ to CP = 4.96, time = 30 min, and catalyst dosage = 4.5 g. In this study, the maximum removal of chlorophenol was 98.9%. In another study, Liu et al.^26^ investigated Fe_2_O_3_-impregnated chitosan beads as an adsorbent to remove As. They reported that the maximum capacity of the adsorbent at a pH of 5 and temperature of 30 °C was 9.355 mg g^−1^.

When the relationship between multiple variables affects the outcome of a process, the use of statistical methods for experimental designs becomes crucial. These methods help select an appropriate design to minimize material consumption, experimental costs, and the time required for experiments^27^. When a combination of several independent variables and their interactions affects the desired responses, RSM becomes an effective tool for process optimization^28^. RSM utilizes an experimental design like the BBD to fit a model through the least squares technique. The adequacy of the proposed model is then evaluated using diagnostic tests provided by analysis of variance (ANOVA). RSM has been commonly employed in various industrial processes for result evaluation and process efficiency assessment^29,30^.

Therefore, the present study deals with the removal efficiency of MB, MG, and Cu using CIST nanocomposite. The removal process in this study was based on RSM, considering influential factors such as pH, concentration, adsorbent amount, and contact time.

## Materials and methods

### Materials and instrumentation

The materials used in the research including ethanol (C_2_H_6_O), cobalt nitrate hexahydrate (Co(NO_3_)_2_·6H_2_O), hydrochloric acid (HCl), titanium (IV) butoxide (C_16_H_36_O_4_Ti), sodium hydroxide (NaOH), ammonia (NH_3_), acetonitrile (C_2_H_3_N), methylene blue (C_16_H_18_ClN_3_S), iron (III) nitrate nonahydrate (Fe(NO_3_)_2_·9H_2_O), potassium nitrate (KNO_3_), tetraethyl orthosilicate (SiC_8_H_20_O_4_), malachite green (C_23_H_25_ClN_2_), and methanol (CH_4_O) were of laboratory purity and obtained from Merck, Germany. Stock solutions (1000 mg L^−1^) of each analyte in water were prepared through double distillation. These stock solutions were utilized to prepare the required concentrations for experiments and standard solutions. NaOH (0.1 M) and HCl (0.1 M) were used to adjust the pH of solutions, and pH measurement was conducted using a pH meter. For adequate mixing and contact between the adsorbent and analyte, a mixer with an intensity of 150 rpm at a temperature of 25 °C was employed. Centrifugation and a magnet were utilized to separate adsorbent particles from the solution during the reaction. The remaining concentration of dyes and metal ions in the samples was measured using UV–Vis spectrophotometry and atomic absorption spectroscopy, respectively. The characteristics of the adsorbent, such as morphology and phase features, were examined using FE-SEM, FTIR, TGA, and XRD.

### ***Synthesis of CoO–Fe***_***2***_***O***_***3***_

Nano-absorbent was synthesized by hydrothermal method. First, 0.62 g of Co(NO_3_)_2_·6H_2_O and 1.71 g of Fe(NO_3_)_2_·9H_2_O were dissolved in 25 mL of double distilled water. Then the mixture was stirred on a stirrer for 1 h at room temperature. Next, NaOH 1 M was slowly added to the desired mixture to adjust its pH to 11. Again, the mixture was stirred for 1 h. Then the solution was transferred to the autoclave and placed under the temperature of 180 °C for 12 h.

### ***Synthesis of CoO–Fe***_***2***_***O***_***3***_***/SiO***_***2***_

In order to synthesize CoO–Fe_2_O_3_/SiO_2_, 1 g of dried CoO–Fe_2_O_3_ powder was mixed with 50 mL of deionized water, and then 150 mL of C_2_H_6_O and 2.5 mL of NH_3_ were added to it. The mixture was dispersed under ultrasonic waves for 30 min. Then, while the solution was stirred under a mechanical stirrer, a mixture of 1.5 mL of SiC_8_H_20_O_4_ and 0.5 mL of NH_3_ was added dropwise to the solution. The reaction was continued for 6 h with continuous stirring at room temperature. Then, the formed particles were washed several times with C_2_H_6_O and deionized water to neutralize them. Finally, the synthesized materials were dried in the oven at a temperature of 60 °C.

### ***Synthesis of CoO–Fe***_***2***_***O***_***3***_***/SiO***_***2***_***/TiO***_***2***_*** (CIST)***

The obtained CoO–Fe_2_O_3_/SiO_2_ nanocomposite was mixed with 125 mL of C_2_H_6_O and subjected to ultrasonic waves for 15 min for homogenization. After that, 12.5 mL of C_16_H_36_O_4_Ti was added dropwise to the mixture under reflux at 45 °C and under constant stirring. The obtained product was washed several times with ethanol and then with distilled water. Finally, it was dried in the oven at 60 °C.

### Experiment design by BBD method

After identifying the influential factors and determining the desired levels through the one-factor-at-a-time method, RSM based on BBD was employed to assess the effect of four independent variables on the removal of MB, MG, and Cu by CIST nanocomposite. For this purpose, the effects of four experimental factors (i.e., pH, concentration, adsorbent amount, and contact time) at three levels (low, medium, and high, denoted by coded values − 1, 0, and + 1, respectively) were investigated. To this end, a set of 27 experiments were designed using the BBD method. The experiments were conducted in triplicate, and after statistical analysis of the results through the ANOVA method, all second-order regression coefficients were estimated. Subsequently, by combining the obtained results and plotting a second-order polynomial equation, the optimal point was precisely determined. Statistical analysis of multivariable equations, determination of second-order regression model coefficients, and the effects of factors on variables were carried out using Design Expert statistical software (Version 12, State-Ease Inc, USA).

### Experimental procedure

This study was conducted at the laboratory scale and in batch mode. The experiments were carried out based on the design provided by RSM. For this purpose, various concentrations of the analytes were prepared in several batches (250 mL). The pH of the samples was studied within the range of 4–10. HCl and NaOH were used to adjust the pH of the samples. In each adsorption experiment, a predetermined amount of adsorbent specified by the software was added to each batch and mixed on a shaker. After the required contact time, the adsorbent was separated from the solution by centrifugation (5 min, 2500 rpm) and using a magnet. Finally, samples were taken from each batch, and the concentration of dyes and metal ions was measured by UV–Vis spectrophotometry and atomic absorption spectroscopy, respectively. The percentage removal of analytes was calculated from Eq. 1.

1 $$\% Removal = \frac{{C_{0} - C_{e} }}{{C_{0} }} \times {1}00$$

where *C*_0_ was the initial concentration (mg L^−1^), and *C*_*e*_ was the concentration at the equilibrium time (mg L^−1^).

## Results and discussion

### Characterization of the CIST nanocomposite

The morphology of CIST nanocomposite was examined through FE-SEM. Fig. S1a provides an image of the adsorbent surface, revealing nearly spherical and uniform magnetic nanocomposite with a particle size of approximately 20 nm, as evident in Fig. S1a. This uniform dispersion and the tiny size of nanoparticles contribute to the creation of a high surface area and a porous magnetic structure in the adsorbent. Additionally, the surface features, such as the present cavities, enhance its capability as an adsorbent.

Thermal stability is a crucial factor affecting the effectiveness of the adsorbent. Thermal analysis of CIST in the temperature range of 30–600 °C is presented in Fig. S1b. These nanocomposites exhibit thermal stability up to 600 °C. CIST shows a slight weight loss at 180 °C, attributed to the negligible weight reduction of metal oxides, with the minimal loss being associated with residual water molecules within the internal sheets and the surface of the nanomaterial.

The phase structure of CIST was examined through XRD analysis. The results are presented in Fig. S1c. In the diffraction pattern of CIST nanocomposite, distinctive peaks at 2θ values of 31.7°, 36.2°, 43.9°, 52.8°, 58.1°, and 65.2° indicate the crystalline structure of these magnetic nanocomposites. Peaks observed at 37.7°, 37.9°, 47.8°, and 54.3° correspond to the presence of titanium dioxide coating. Higher peaks at 2θ around 23.6° are also associated with the silica coating^31,32^. The particle size was determined to be 30 nm by Scherrer’s equation.

The FT-IR spectrum of CIST in the wavelength range of 400–3400 cm^−1^ is illustrated in Fig. S1d. Spectrum analysis reveals a prominent peak at approximately 603.5 cm^−1^, attributed to the presence of Co–O and Fe–O bonds. The peak observed at 1623 cm^−1^ is associated with water molecules in the inner layers of the nanoparticle structure, while the broad peak at 3284.6 cm^−1^ is indicative of stretching vibrations of O–H in silanol groups and water molecules. The absorption peak at 1099.5 cm^−1^ corresponds to the stretching vibration of Si–O–Si bonds, and peaks around 756.1 cm^−1^ indicate stretching and bending vibrations of Ti–O–Si, indicating the presence of titanium dioxide and silica coatings on the surface of CIST nanocomposite^33,34^.

### Box–Behnken design

After selecting the effective factors, the BBD method was used to optimize the removal of MB, MG and Cu under the conditions of adsorbent amount, pH, analyte concentration and contact time. Each of the independent variables was defined at three levels: low (− 1), medium (0), and high (+ 1), based on coded values (Table 1).

**Table 1:** The BBD matrix.

Variables	Symbol	Unit	Range and levels		
			− 1	0	+ 1
Nanocomposite amount	A	g	0.01	0.02	0.03
pH of the solution	B	–	4	7	10
Analyte concentration	C	Mg L^−1^	20	40	60
Contact time	D	min	5	10	15

The designed experiment included 27 different experiments, and the removal efficiency was considered as the response surface to the variables. In Table S1, the results of the conducted experiments using BBD, along with the obtained removal percentages and predicted removal percentages, are presented. In the RSM approach, a model is defined for each dependent variable, expressing the main and interaction effects of factors on each variable separately. In this study, a second-order polynomial experimental model was used to investigate the four experimental variables. The response of the experimental system was based on Eq. 2.

2 $${\text{Y}} = \beta_{0} + \mathop \sum \limits_{i = 1}^{k} \beta_{i} X_{i} + \mathop \sum \limits_{i = 1}^{k} \beta_{ii} X_{i}^{2} + \mathop \sum \limits_{i \le j}^{k} \mathop \sum \limits_{j}^{k} \beta ijX_{i} X_{j} + e$$

In this equation, *Y* was removal efficiency, *X*_*i*_, and *X*_*j*_ were the coded values of the factors, *β*_0_ was the constant, and *β*_*i*_, *β*_*j*_, and *β*_*ij*_ were the regression coefficients calculated by the software^35,36^. After selecting the appropriate mathematical model, statistical analysis of the data, examination of the adequacy of the experimental model, and optimization plot drawing were performed using the Design Expert software. Statistical data analysis was performed to assess the appropriateness of the applied experimental model. The ANOVA results for the polynomial response surface experimental model are presented in Tables 2, 3 and 4. As can be seen, the high quantities of the F-value and the obtained Probability ˃ F values (< 0.0001) denote the significance of the applied model^37^. Additionally, as shown in Tables 2, 3 and 4, the non-significant Lack of Fit mean squares indicate the adequacy of the second-degree model and the absence of other relationships affecting the removal efficiency. In conclusion, it can be inferred that the results obtained with the applied experimental model are in complete agreement.

**Table 2:** ANOVA for removal of MB.

Source	Sum of squares	Degree of freedom	Mean square	F-value	*p*-value	
Model	11,507.80	14	821.99	779.99	< 0.0001	significant
A	887.69	1	887.69	842.34	< 0.0001	
B	145.81	1	145.81	138.36	< 0.0001	
C	64.31	1	64.31	61.02	< 0.0001	
D	152.80	1	152.80	144.99	< 0.0001	
AB	214.92	1	214.92	203.94	< 0.0001	
AC	2469.59	1	2469.59	2343.42	< 0.0001	
AD	45.43	1	45.43	43.11	< 0.0001	
BC	760.10	1	760.10	721.27	< 0.0001	
BD	294.98	1	294.98	279.91	< 0.0001	
CD	394.22	1	394.22	374.08	< 0.0001	
A^2^	3221.76	1	3221.76	3057.15	< 0.0001	
B^2^	3278.39	1	3278.39	3110.89	< 0.0001	
C^2^	31.62	1	31.62	30.00	< 0.0001	
D^2^	1345.55	1	1345.55	1276.80	< 0.0001	
Residual	14.75	14	1.05			
Lack of fit	10.44	10	1.04	0.9692	0.5627	Not significant
Pure error	4.31	4	1.08			
Cor total	11,522.55	28				
R^2^ = 0.9987	Adjusted R^2^ = 0.9974	Predicted R^2^ = 0.9942		Adeq-Precision = 92.75

**Table 3:** ANOVA for removal of MG.

Source	Sum of squares	Degree of freedom	Mean square	F-value	*p*-value	
Model	6935.53	14	495.39	174.32	< 0.0001	Significant
A	157.25	1	157.25	55.33	< 0.0001	
B	114.76	1	114.76	40.38	< 0.0001	
C	255.49	1	255.49	89.90	< 0.0001	
D	139.81	1	139.81	49.20	< 0.0001	
AB	361.19	1	361.19	127.09	< 0.0001	
AC	1636.61	1	1636.61	575.89	< 0.0001	
AD	35.64	1	35.64	12.54	0.0033	
BC	764.80	1	764.80	269.12	< 0.0001	
BD	72.34	1	72.34	25.45	0.0002	
CD	118.92	1	118.92	41.84	< 0.0001	
A^2^	2181.87	1	2181.87	767.75	< 0.0001	
B^2^	1017.84	1	1017.84	358.16	< 0.0001	
C^2^	155.21	1	155.21	54.62	< 0.0001	
D^2^	1219.30	1	1219.30	429.04	< 0.0001	
Residual	39.79	14	2.84			
Lack of fit	27.44	10	2.74	0.8890	0.6018	Not significant
Pure error	12.35	4	3.09			
Cor total	6975.31	28				
R^2^ = 0.9943	Adjusted R^2^ = 0.9886	Predicted R^2^ = 0.9746		Adeq-Precision = 46.03

**Table 4:** ANOVA for removal of Cu.

Source	Sum of Squares	Degree of freedom	Mean Square	F-value	*p*-value	
Model	6290.34	14	449.31	337.80	< 0.0001	Significant
A	118.13	1	118.13	88.81	< 0.0001	
B	191.60	1	191.60	144.05	< 0.0001	
C	733.99	1	733.99	551.83	< 0.0001	
D	381.83	1	381.83	287.07	< 0.0001	
AB	9.30	1	9.30	6.99	0.0192	
AC	1204.44	1	1204.44	905.53	< 0.0001	
AD	76.04	1	76.04	57.17	< 0.0001	
BC	343.36	1	343.36	258.15	< 0.0001	
BD	112.68	1	112.68	84.71	< 0.0001	
CD	2.89	1	2.89	2.17	0.1626	
A^2^	2268.60	1	2268.60	1705.61	< 0.0001	
B^2^	1222.59	1	1222.59	919.18	< 0.0001	
C^2^	80.89	1	80.89	60.82	< 0.0001	
D^2^	554.27	1	554.27	416.72	< 0.0001	
Residual	18.62	14	1.33			
Lack of fit	9.93	10	0.9933	0.4573	0.8651	Not significant
Pure error	8.69	4	2.17			
Cor total	6308.96	28				
R^2^ = 0.9970	Adjusted R^2^ = 0.9941	Predicted R^2^ = 0.9888		Adeq-Precision = 62.78		

As shown in Tables 2, 3 and 4, the regression coefficients for the linear fit R^2^ were 99.87%, 99.43%, and 99.70% for MB, MG, and Cu, respectively. This indicates that over 99.4% of the variations in the removal are accounted for by the used dependent variables, and less than 2% of these variations cannot be justified by the experimental model. Moreover, the adjusted R^2 ^˃ 0.98 for all three analytes was very close to R^2^, implying a good fit for the model. Therefore, the high values of R^2^ and adjusted R^2^ provide evidence for the appropriateness of the applied experimental model in this study. After ensuring the adequacy of the employed polynomial experimental model, multiple regression analysis using Design Expert software was conducted to determine the coefficients of the regression equation. Finally, solving the second-degree multivariable equation was employed to find the optimal point. Based on the regression analysis results and incorporating the coefficients into the second-degree polynomial equation, Eqs. 3–5 can be utilized for the optimal prediction of MB, MG, and Cu removal.

3 $$\begin{aligned} \% {\text{Removal}}\;{\text{of}}\;{\text{MB}} & = + {79}.{28} + {8}.{6}0{\text{A}} - {3}.{\text{48B}} - {2}.{\text{31C}} - {3}.{\text{56D}} - {7}.{\text{33AB}} - {24}.{\text{84AC}} - {3}.{\text{37AD}}\;{3}.{\text{78BC}} \\ & \quad - {8}.{\text{58BD}} - {9}.{\text{92CD}} - {22}.{\text{28A}}^{2} - {22}.{\text{48B}}^{2} - {2}.{2}0{\text{C}}^{2} - {14}.{4}0{\text{D}}^{2} \\ \end{aligned}$$

4 $$\begin{aligned} \% \;{\text{Removal}}\;{\text{of}}\;{\text{MG}} = + {9}0.{38} + {3}.{\text{62A}} + {3}.0{\text{9B}} + {4}.{\text{61C}} - {3}.{\text{41D}} - {9}.{5}0{\text{AB}} - {2}0.{\text{22AC}} + {2}.{\text{98AD}} \\ & \quad + {13}.{\text{82BC}} - {4}.{\text{25BD}} - {5}.{\text{45CD}} - {18}.{\text{34A}}^{{2}} - {12}.{\text{52B}}^{{2}} - {4}.{\text{89C}}^{{2}} - {13}.{\text{71D}}^{{2}} \\ \end{aligned}$$

5 $$\begin{aligned} \% {\text{Removal}}\;{\text{of}}\;{\text{Cu}} & = + {88}.{45} + {3}.{\text{13A}} - {3}.{\text{99B}} + {7}.{\text{82C}} - {5}.{\text{64D}} - {1}.{\text{52AB}} - {17}.{\text{35AC}} + {4}.{\text{36AD}} + {9}.{\text{26BC}} \\ & \quad - {5}.{3}0{\text{BD}} - 0.{\text{85CD}} - {18}.{7}0{\text{A}}^{{2}} - {13}.{\text{72B}}^{{2}} - {3}.{\text{53C}}^{{2}} - {9}.{\text{24D}}^{{2}} \\ \end{aligned}$$

In this equation, A was the amount of adsorbent (g), B was pH, C was concentration (mg L^−1^), and D was time (min).

In addition to the mentioned criteria for evaluating the accuracy of the selected model, the difference between predicted and experimental responses (residuals) has been graphically utilized to assess the model’s precision. Residuals are considered as the deviations not captured by the model. The residual plots for evaluating the normal distribution of residuals are presented in Figs. S2a–c. The points in the residual normality plots form a straight line, confirming that residuals are normally distributed. Figs. S2d–f depict the predicted values against the actual values of the studied variables for MB, MG, and Cu. As observed, there is sufficient agreement between the actual data and the model-derived data, indicating the suitability of the selected model for the dataset.

### 3D response surface

**Figure 1:**
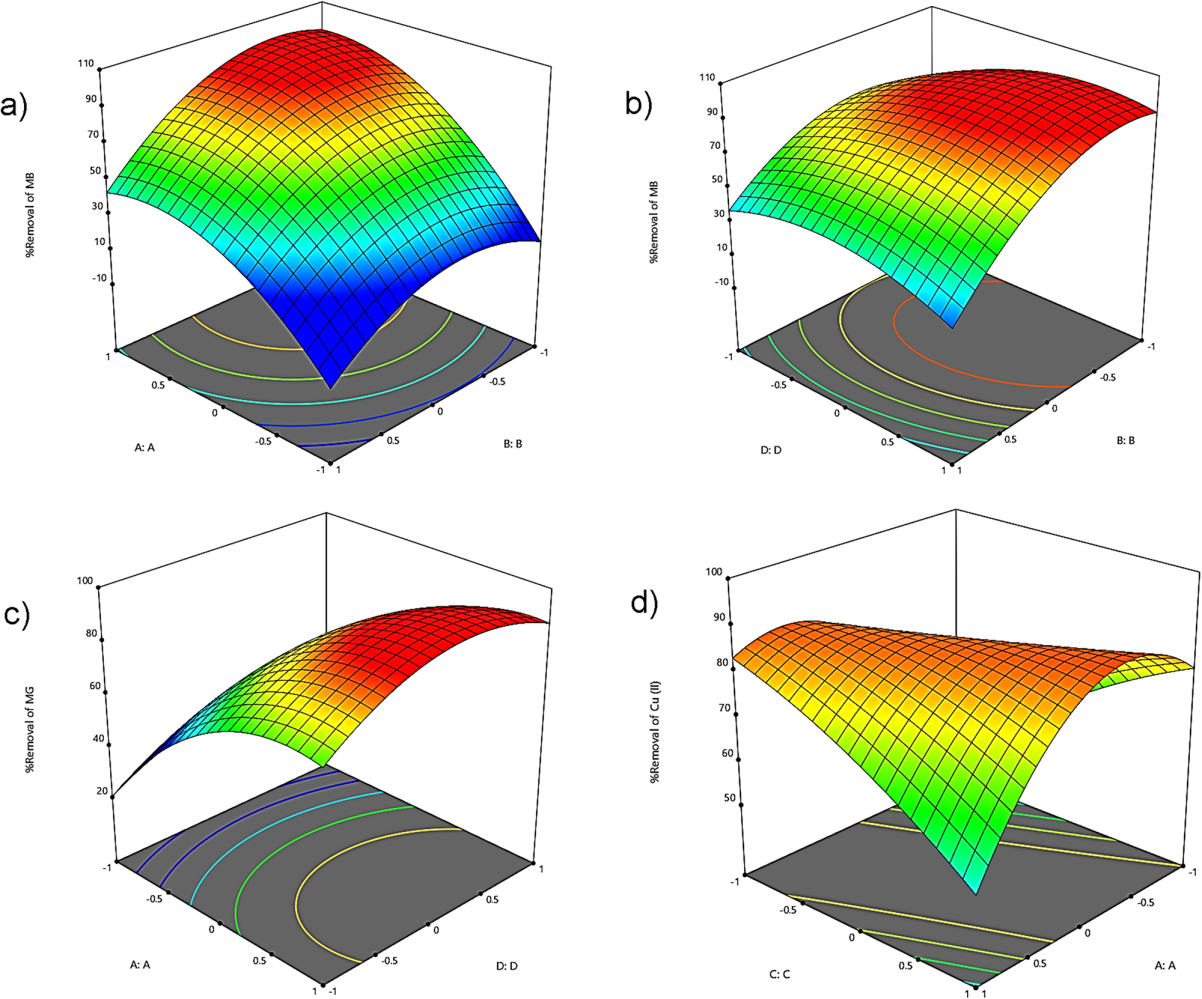
The three-dimensional (3D) plots.

The adsorbent mass is one of the influential factors in surface adsorption examined in the experiments. The results obtained from this study (Fig. 1a) indicate that by increasing the adsorbent mass from 0.01 to 0.03 g, the removal efficiency of MB increases. This enhancement in MB removal efficiency was observed at a contact time of 10 min and pH of 6 by varying the mass of CIST. The reason for the increased MB removal efficiency is that the higher adsorbent mass leads to an increase in the number of active adsorption sites in the solution. Consequently, the increased number of active sites enhances the contact surface between the adsorbent and the pollutant, resulting in improved pollutant removal efficiency. Similar results were obtained in the studies by Qu et al.^38^ and Kadhom et al.^39^.

Another influential factor in the adsorption process is pH. This is because it induces a change in the ion state of the analyte, ionization, and surface charge of the adsorbent, affecting the interaction between the adsorbent and the adsorbate substance. In this study, it was observed (Fig. 1b) that the removal efficiency was low at very low pH levels. The reason for the low efficiency at acidic pH is creating a positive charge on the adsorbent, leading to an electrostatic repulsive force between the adsorbent and MB. Additionally, the concentration of hydrogen ions in the solution increases under acidic conditions, competing with MB for adsorption sites, resulting in decreased MB removal efficiency. However, as the pH increases, the concentration of hydrogen ions decreases, leading to increased MB adsorption. The study demonstrated that with an increase in pH, the removal efficiency of MB also increases. The removal efficiency of MB significantly increases by changing the pH from 4 to 6. Similar results were obtained in the studies by Jawad and Surip^40^ and Mehmandost et al.^41^, indicating the similarity of the present study with their findings.

The required time for the interaction between the adsorbent and the adsorbate is a crucial factor that needs to be investigated in adsorption studies. The results of this study (Fig. 1c) demonstrated that with an increase in contact time, the removal efficiency of MG increases. This is attributed to the fact that, with an extended contact time, dye molecules have more opportunities to interact with the adsorbent surface. Initially, the adsorption capacity increases with time, but after a while, it reaches a relatively constant level, indicating the attainment of equilibrium. The initial increase in adsorption rate is due to the availability of more active sites and functional groups during the early stages. The study results revealed that the equilibrium time for this adsorbent is approximately 10 min. In a study conducted by Azam et al. in^42^ on the removal of Cr and Cd using treated date seeds, it was found that the removal efficiency of Cr and Cd increases with contact time. Moreover, the highest adsorption occurs within the first 5 h. A similar outcome was observed in the study by Shojaei et al. in^43^, where they found an increase in removal efficiency with prolonged contact time until reaching equilibrium, which took approximately 20 min in their study. Afterward, no significant changes were observed.

Another factor investigated in this study was the pollutant concentration. The effect of Cu concentration on the removal efficiency was examined in the range of 20–60 mg L^−1^, and the results are depicted in Fig. 1d. The study results revealed a decrease in removal efficiency with an increase in concentration from 20 to 60 mg L^−1^. The reason for the reduction in removal efficiency at higher concentrations is that the active adsorption sites on the adsorbent's surface become saturated with the pollutant. As a result, the collision between analyte ions and active sites decreases. The findings of this part of the study align with the studies conducted by Mustafa et al.^44^ and Gajera et al.^45^.

### Optimization

In the removal process of MB, MG, and Cu, achieving the maximum removal was the primary objective in the experimental analyses. The optimal operational conditions were determined using numerical optimization techniques. Initially, optimization objectives, response surfaces, and independent variables were defined. The desirability function method was employed to obtain the best responses. The optimal removal conditions included an adsorbent dosage of 0.025 g, pH of 6, a concentration of 20 mg L^−1^, and a contact time of 10 min. Under these conditions, the removal efficiency for MB, MG, and Cu was 98.01%, 93.06%, and 88.26%, respectively (Table 5).

**Table 5:** Optimum variables of removal of pollutants (n = 3).

Variables				%Removal of MB		%Removal of MG		%Removal of Cu	
A	B	C	D	Experimental	Predicted	Experimental	Predicted	Experimental	Predicted
0.025	6	20	10	98.01 ± 2.0	97.49	93.06 ± 1.5	94.50	88.26 ± 1.8	86.25

### Desorption studies

**Figure 2:**
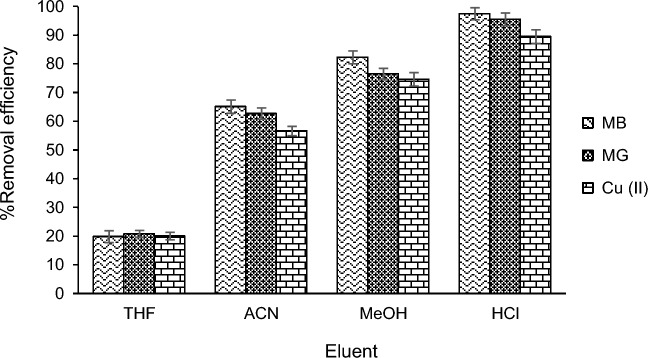
The effect of eluent solvent on the desorption process.

The appropriate eluent must be capable of thoroughly washing the adsorbed analyte from the adsorbent, allowing the adsorbent to be ready for reuse in experiments. In this study, tetrahydrofuran (THF), acetonitrile (ACN), methanol (MeOH), and hydrochloric acid (HCl) were employed as eluents. After each experiment, the adsorbent was separated from the solution using a magnet and placed in contact with the desired solvents to examine the desorption efficiency. The results of the eluent investigation on the analyte desorption efficiency are depicted in Fig. 2. According to the results in Fig. 2, HCl was suitable for desorbing MB, MG, and Cu from the surface of CIST, and it was utilized as the eluent in the experiments.

### The determination of zeta potential

The point at which the positive and negative charges are equal is the zero point of charge (pH_zpc_) for the adsorbent. At pH values higher than the pH_zpc_, the surface charge potential on the adsorbent is negative, and at pH values lower than the pH_zpc_, the surface charge potential is positive. To determine the pH_zpc_, 50 mL of 0.01 M KNO_3_ was added to several beakers. Subsequently, the solution pH in the range of 2–10 was adjusted using 0.1 M HCl and 0.1 M NaOH. Next, 0.2 g of the adsorbent was added to each specified beaker and left to equilibrate for 24 h. The final solution pH was then measured using a pH meter, and a plot of initial pH versus final pH was generated. The intersection point of the two curves was identified as the pH_zpc_. The results of investigating the pH_zpc_ for the CIST adsorbent are presented in Fig. S3. According to Fig. S3, the pH_zpc_ for the adsorbent was determined to be 5.1.

### The reusability of CIST nanocomposite

**Figure 3:**
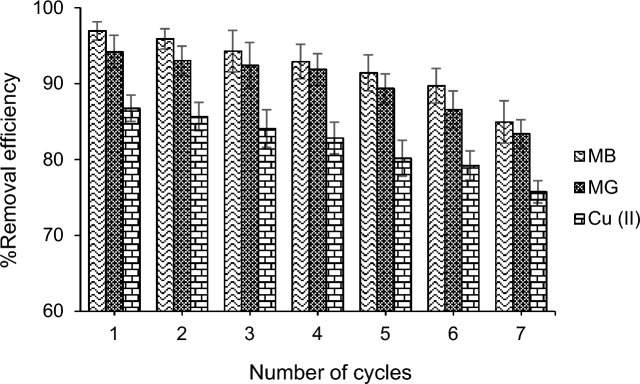
Influence of the reuse cycle of CIST nanocomposite.

From a practical perspective, the recovery or disposal of pollutants from the adsorbent surface in the surface adsorption process is beneficial. The recovery and reuse of the adsorbent are crucial factors for evaluating its performance in practical applications. Therefore, after adsorbing MB, MG, and Cu from the aqueous solution with the CIST adsorbent, the adsorbent was recovered by washing with HCl and reused. The results of consecutive adsorption–desorption cycles for MB, MG, and Cu using the CIST adsorbent are shown in Fig. 3. According to the obtained results, the percentage of analyte desorption decreased by 12% until the seventh cycle. These results demonstrate the significant stability of the adsorbent and the non-destruction of the synthesized nanocomposite structure over several usage cycles. This indicates the economic viability of synthesizing and employing this adsorbent in treating wastewater containing MB, MG, and Cu.

### Real samples analysis

To assess the efficiency of the proposed method in removing MB, MG, and Cu from real water samples, several water samples were utilized, including tap water, fish farm water, and wastewater. The procedure involved adding specified amounts of analytes to a fixed volume of water samples to prepare them for analysis. The desired samples were analyzed using the proposed method and under optimal conditions, with each experiment repeated five times. The obtained results are presented in Table 6. The results demonstrated that the removal efficiency of MB, MG, and Cu for real water samples ranged from 81.69 to 98.18%. The findings indicated that the sample matrix had a limited impact on the removal of MB, MG, and Cu from real water samples.

**Table 6:** Results of real samples under the optimum conditions (n = 5).

Analytes	Samples	%Removal ± %RSD
MB	Tap water	96.65 ± 2.5
	Fish farm	98.18 ± 1.7
	Wastewater	92.96 ± 2.9
MG	Tap water	93.57 ± 2.6
	Fish farm	95.87 ± 2.0
	Wastewater	90.57 ± 2.7
Cu	Tap water	86.10 ± 1.7
	Fish farm	88.04 ± 2.2
	Wastewater	81.69 ± 1.6

### Comparison CIST nanocomposite with other adsorbents

According to the results of the present study, CIST nanocomposite can be used as an adsorbent for the effective removal of MB, MG, and Cu from water solutions. Therefore, the performance of CIST nanocomposite was compared with other adsorbents used to remove MB, MG, and Cu (Table 7). As shown in this table, CIST nanocomposite was better or comparable to other adsorbents in removing MB, MG, and Cu. Also, using RSM leads to a reduction in process time, a reduction in the number of tests, and a reduction in the use of materials.

**Table 7:** Comparison of the CIST nanocomposite with other adsorbents.

Adsorbent	Analyte	pH	Adsorbent amount	Time	Result	References
Zeolite NaA	MB	7	1 g	1 h	53.5 mg g^−1^	^46^
Sugar scum	MB	8	4 g	2 h	49.74%	^47^
Wood millet carbon	MB	7	0.25 g	18 min	99%	^48^
Algerian kaolin	MB	6	0.5 g	120 min	52.76 mg g^−1^	^49^
Copper nanowires loaded on activated carbon	MG	5	0.1 g	20 min	434.80 mg g^−1^	^50^
walnut shell	MG	7	0.03 g	120 min	90.8 mg g^−1^	^51^
Zinc sulfide	MG	8	0.08 g	120 min	98.30%	^52^
Halloysite nanotubes	MG	9	0.2 g	30 min	99.60%	^53^
Hyacinth roots	Cu	6	14.08 g	150 min	21.79 mg g^−1^	^54^
Lignocellulosic waste N-CDs	Cu	7	100 mg	30 min	26.95 mg g^−1^	^55^
Humic acid modified nano-hydroxyapatite	Cu	5.5	2.2 g	8 h	97.68%	^56^
Chitosan-montmorillonite hydrogel	Cu	6	80 mg	2 h	132.74 mg g^−1^	^57^
CIST nanocomposite	MB	6	0.014 g	10 min	98.01%	Our work
CIST nanocomposite	MG	6	0.014 g	10 min	93.06%	Our work
CIST nanocomposite	Cu	6	0.025 g	10 min	88.26%	Our work

## Conclusion

In the present study, a CIST nanocomposite was synthesized and characterized by various techniques (XRD, TGA, FTIR, pH_pzc_, and SEM). Additionally, the influential factors affecting the removal, such as contact time, concentration, pH, and the amount of adsorbent, were investigated using RSM based on BBD. The removal efficiency of MB, MG, and Cu was examined under optimal conditions (i.e., pH of 6, contact time of 10 min, adsorbent amount of 0.025 g, and concentration of 20 mg L^−1^). The removal percentages of MB, MG, and Cu from real wastewater ranged from 81.69 to 98.18%. The reusability assessment of CIST nanocomposite demonstrated its suitable stability for repeated use in removing MB, MG, and Cu. According to the results obtained in this study, the synthesized nanocomposite can be considered a renewable adsorbent with significant impacts on wastewater treatment containing MB, MG, and Cu, making it an efficient material for removing these pollutants.

### Supplementary Information

Supplementary Information.

## Data Availability

The authors declare that data supporting the findings of this study are available within the paper.
