# Supplementary material for: Optimization by Box–Behnken design for environmental contaminants removal using magnetic nanocomposite
Source: Sci Rep. 2024 Mar 23;14:6950. doi: 10.1038/s41598-024-57616-8 (PMC10960869; doi:10.1038/s41598-024-57616-8)
Supplement: Supplementary file 1 — Supplementary Information. [file 41598_2024_57616_MOESM1_ESM.docx]

**Supplementary Material**

**Title**

**Optimization by Box-Behnken design for environmental contaminants removal using magnetic nanocomposite**

**Tables captions**

**Table S1.** The results of BBD.

**Figures captions**

**Fig. S1.** a) FE-SEM, b) TGA, c) XRD, and d) FTIR of CIST nanocomposite.

**Fig. S2.** a) Normal plot of residuals for a) MB, b) MG, and c) Cu; Plot of predicted values versus actual values for d) MB, e) MG, and f) Cu.

**Fig. S3.** The pH_pzc_ of CIST nanocomposite.

**Table S1.** The results of BBD.

|  | Variables | | | | %Removal of MB | | %Removal of MG | | %Removal of Cu | |
| --- | --- | --- | --- | --- | --- | --- | --- | --- | --- | --- |
| Run | A | B | C | D | Experimental | Predicted | Experimental | Predicted | Experimental | Predicted |
| 1 | 0 | -1 | -1 | 0 | 73.94 | 74.18 | 79.59 | 79.08 | 76.03 | 76.63 |
| 2 | 0 | 0 | 1 | 1 | 47.87 | 46.87 | 68.40 | 67.53 | 77.70 | 77.01 |
| 3 | -1 | 1 | 0 | 0 | 29.70 | 29.76 | 67.61 | 68.49 | 50.80 | 50.41 |
| 4 | 0 | 1 | 1 | 0 | 62.21 | 62.58 | 94.50 | 94.50 | 84.83 | 84.28 |
| 5 | 1 | 0 | -1 | 0 | 91.24 | 90.56 | 85.41 | 86.38 | 80.29 | 78.89 |
| 6 | 0 | 0 | -1 | 1 | 71.36 | 71.35 | 68.57 | 69.20 | 62.21 | 63.07 |
| 7 | 0 | 1 | -1 | 0 | 38.76 | 39.64 | 59.17 | 57.61 | 49.96 | 50.11 |
| 8 | -1 | 0 | -1 | 0 | 24.79 | 23.66 | 39.18 | 38.69 | 38.99 | 37.91 |
| 9 | -1 | 0 | 0 | -1 | 33.81 | 34.20 | 61.50 | 61.11 | 67.41 | 67.37 |
| 10 | 1 | -1 | 0 | 0 | 54.31 | 53.94 | 70.34 | 69.54 | 64.11 | 64.68 |
| 11 | 1 | 0 | 1 | 0 | 35.41 | 36.23 | 54.23 | 55.15 | 58.98 | 59.83 |
| 12 | 0 | 0 | 0 | 0 | 80.06 | 79.29 | 89.05 | 90.38 | 86.93 | 88.45 |
| 13 | 0 | 0 | -1 | -1 | 57.94 | 58.63 | 64.17 | 65.12 | 71.77 | 72.65 |
| 14 | 1 | 0 | 0 | -1 | 58.65 | 58.14 | 64.72 | 62.38 | 65.20 | 64.93 |
| 15 | 1 | 1 | 0 | 0 | 31.79 | 32.31 | 55.36 | 56.72 | 53.47 | 53.64 |
| 16 | -1 | 0 | 0 | 1 | 32.67 | 33.80 | 46.48 | 48.31 | 47.04 | 47.37 |
| 17 | 0 | 0 | 0 | 0 | 78.77 | 79.29 | 92.39 | 90.38 | 86.77 | 88.45 |
| 18 | 0 | 0 | 1 | -1 | 74.16 | 73.86 | 85.81 | 85.26 | 90.66 | 89.99 |
| 19 | 0 | 0 | 0 | 0 | 78.95 | 79.29 | 91.61 | 90.38 | 89.42 | 88.45 |
| 20 | 0 | 1 | 0 | -1 | 51.97 | 51.07 | 74.29 | 74.90 | 71.95 | 72.43 |
| 21 | 0 | -1 | 0 | 1 | 50.32 | 50.91 | 62.07 | 61.89 | 69.86 | 69.14 |
| 22 | 0 | -1 | 0 | -1 | 40.24 | 40.87 | 58.49 | 60.21 | 70.18 | 69.81 |
| 23 | 0 | -1 | 1 | 0 | 42.25 | 41.98 | 59.61 | 60.66 | 73.84 | 73.74 |
| 24 | 0 | 1 | 0 | 1 | 27.70 | 26.76 | 60.86 | 59.57 | 50.40 | 50.54 |
| 25 | 1 | 0 | 0 | 1 | 44.03 | 44.26 | 61.64 | 61.52 | 62.27 | 62.36 |
| 26 | -1 | 0 | 1 | 0 | 68.35 | 68.73 | 88.91 | 88.37 | 87.09 | 88.26 |
| 27 | -1 | -1 | 0 | 0 | 22.90 | 22.08 | 44.58 | 43.30 | 55.34 | 55.36 |

| 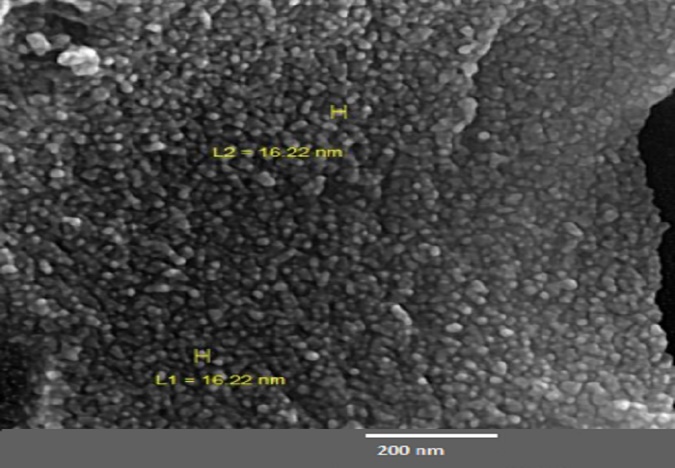  b)  a) | 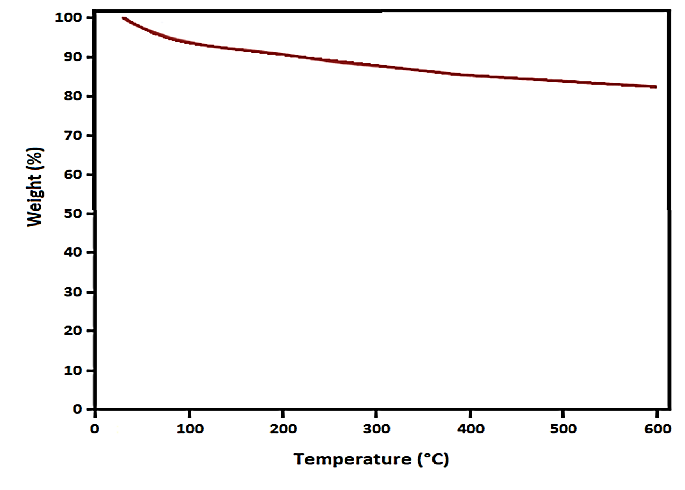 |
| --- | --- |
| 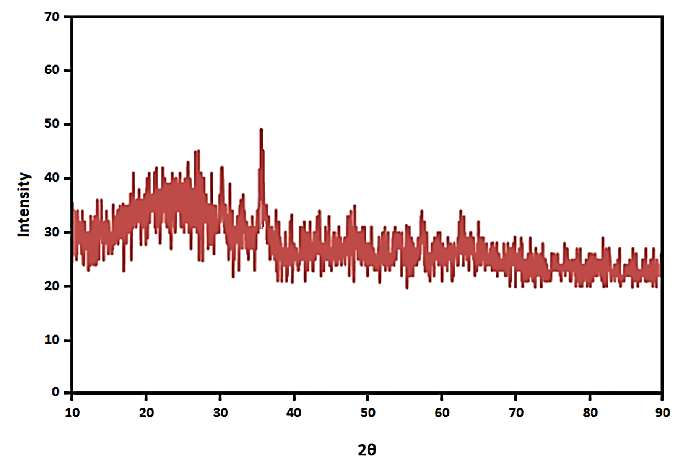  d)  c) | 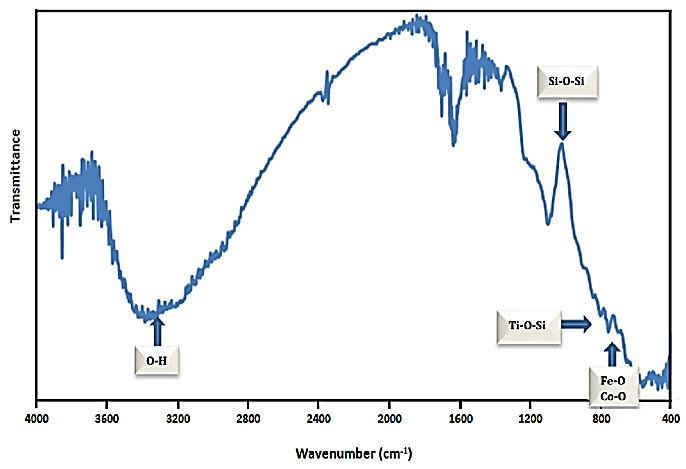 |
| **Fig. S1.** a) FE-SEM, b) TGA, c) XRD, and d) FTIR of CIST nanocomposite. | |

| 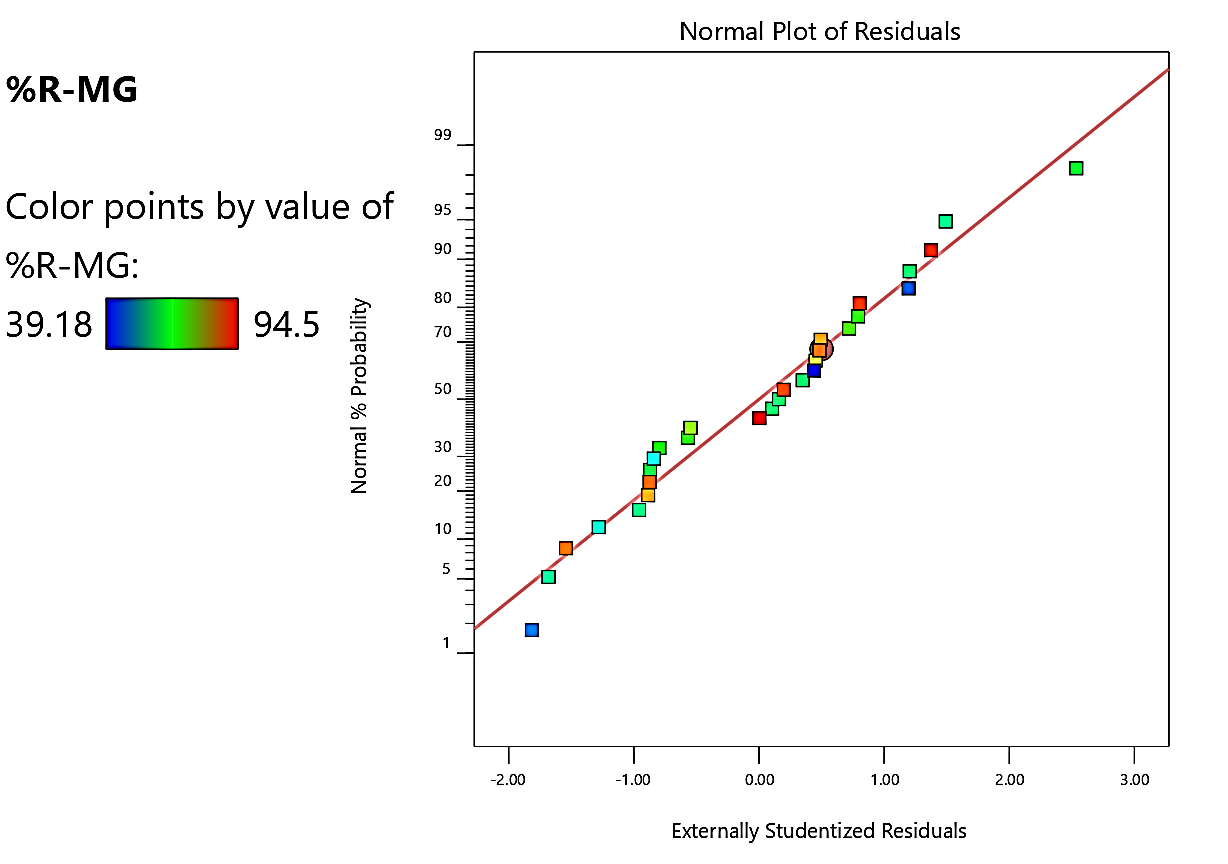 | 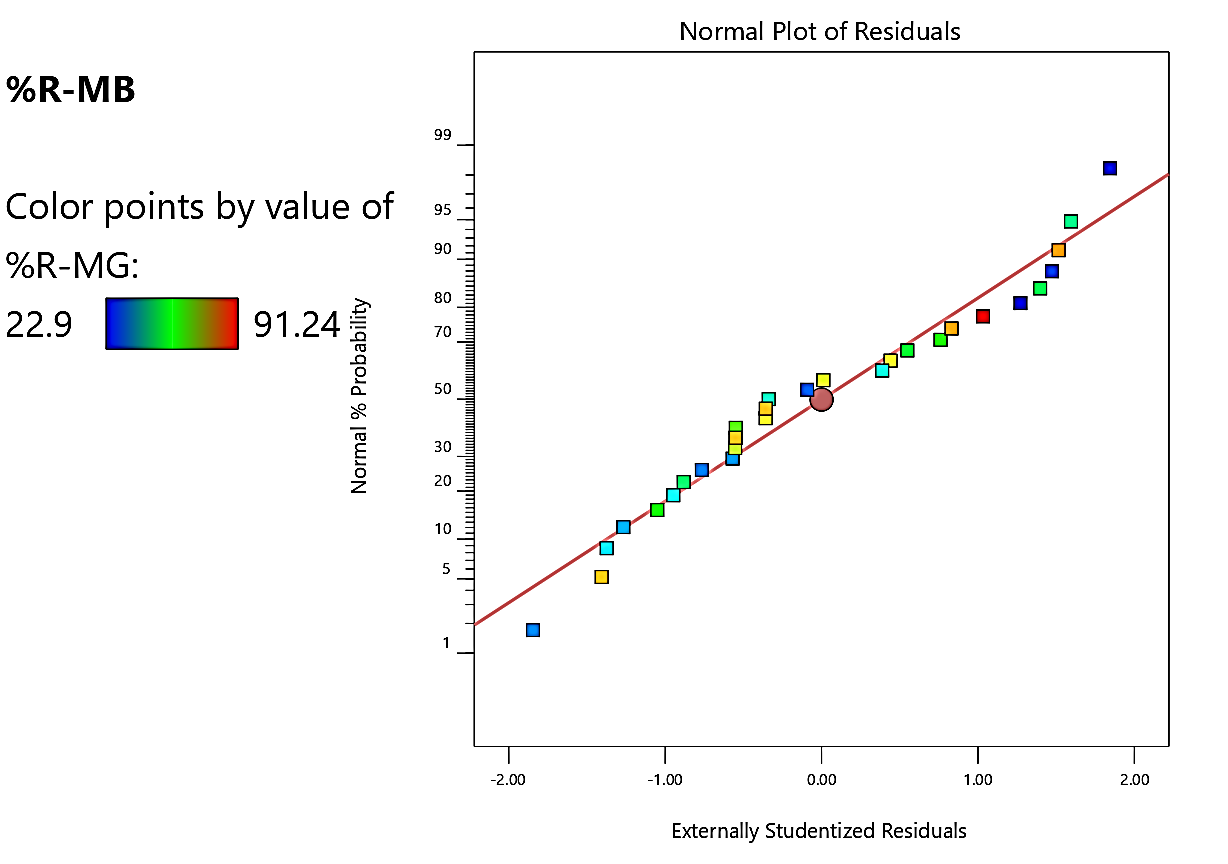  b)  a) |
| --- | --- |
| 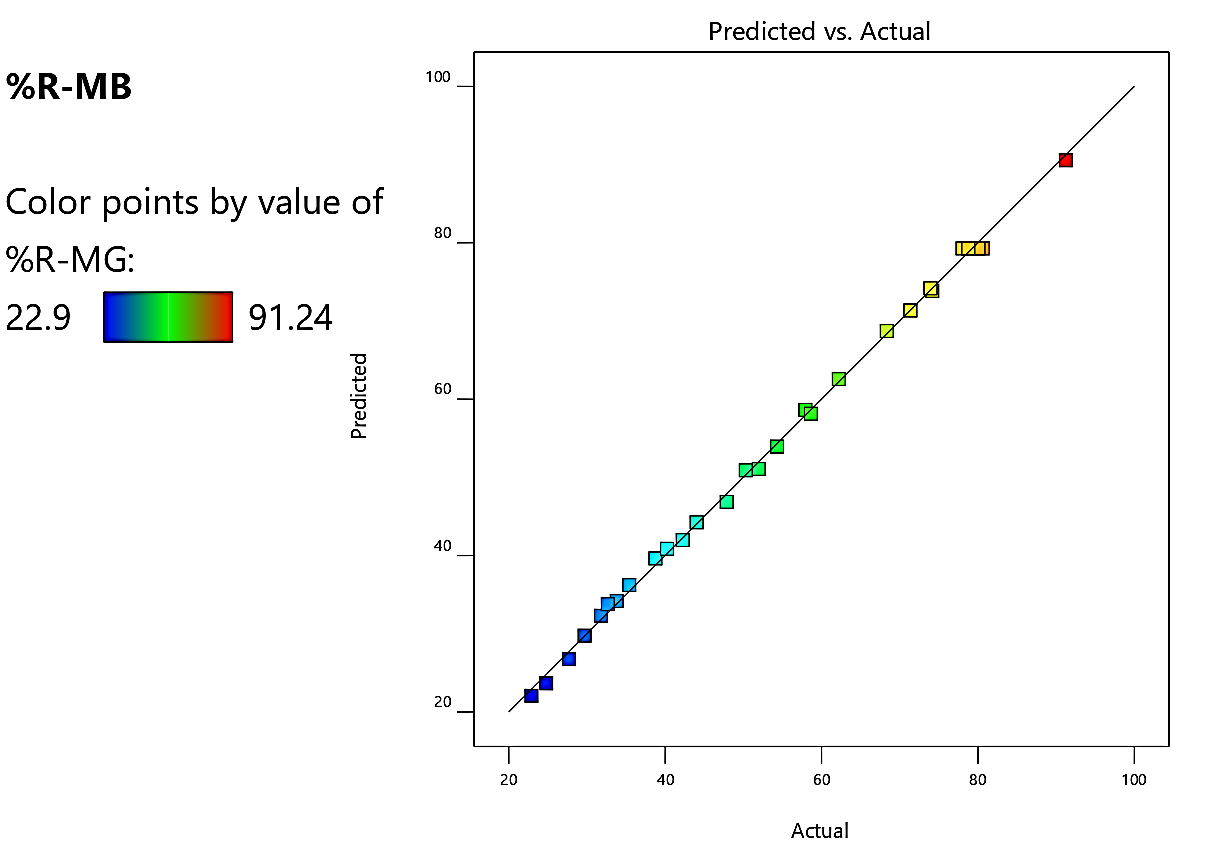 | 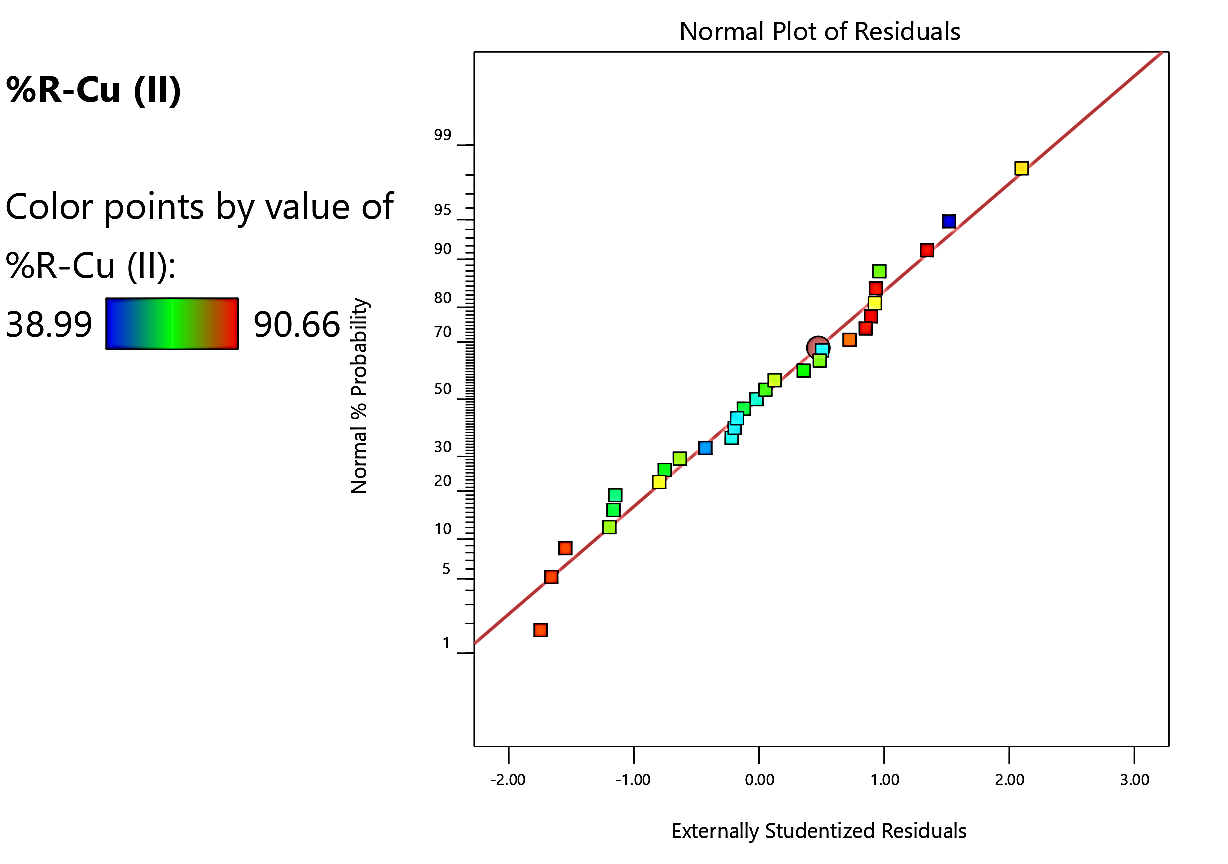  d) |
| 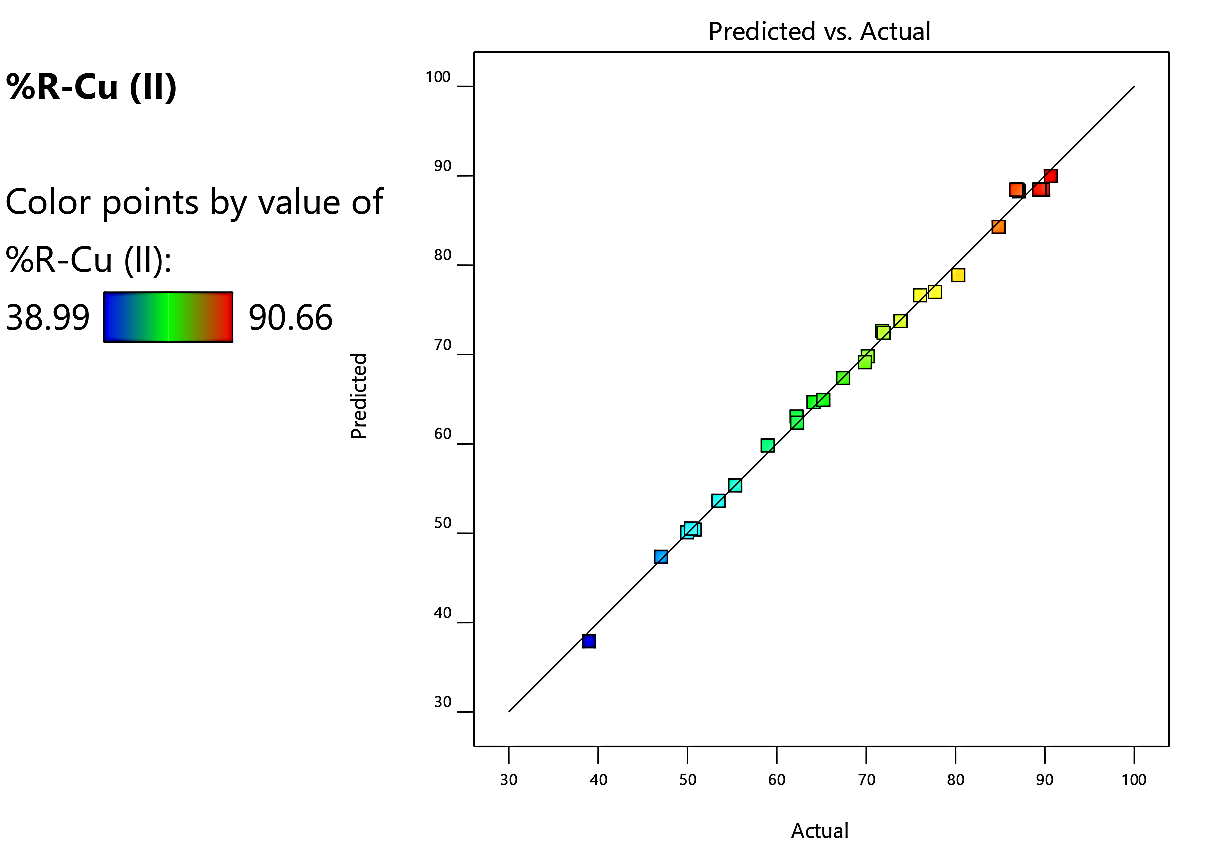 | 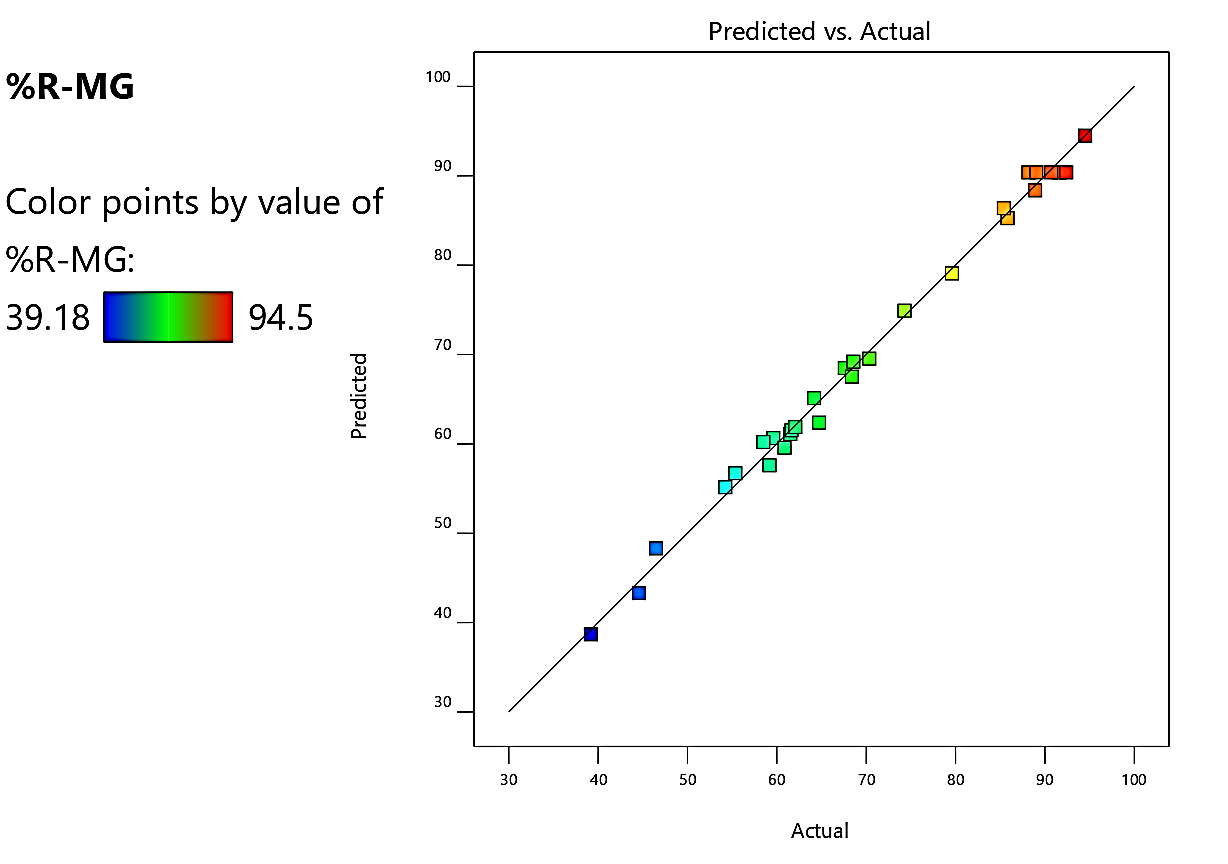  f) |
| **Fig. S2.** a) Normal plot of residuals for a) MB, b) MG, and c) Cu; Plot of predicted values versus actual values for d) MB, e) MG, and f) Cu. | |

e)

c)

**Fig. S3.** The pH_pzc_ of CIST nanocomposite.
